# Are All Beliefs Equal? Implicit Belief Attributions Recruiting Core Brain Regions of Theory of Mind

**DOI:** 10.1371/journal.pone.0106558

**Published:** 2014-09-26

**Authors:** Ágnes Melinda Kovács, Simone Kühn, György Gergely, Gergely Csibra, Marcel Brass

**Affiliations:** 1 Cognitive Development Centre, Central European University, Budapest, Hungary; 2 Max Planck Institute for Human Development, Center for Lifespan Psychology, Berlin, Germany; 3 Department of Experimental Psychology and Ghent Institute of Functional and Metabolic Imaging, Ghent University, Ghent, Belgium; 4 Behavioural Science Institute, Radboud University, Nijmegen, The Netherlands; University College London, United Kingdom

## Abstract

Humans possess efficient mechanisms to behave adaptively in social contexts. They ascribe goals and beliefs to others and use these for behavioural predictions. Researchers argued for two separate mental attribution systems: an implicit and automatic one involved in online interactions, and an explicit one mainly used in offline deliberations. However, the underlying mechanisms of these systems and the types of beliefs represented in the implicit system are still unclear. Using neuroimaging methods, we show that the right temporo-parietal junction and the medial prefrontal cortex, brain regions consistently found to be involved in explicit mental state reasoning, are also recruited by spontaneous belief tracking. While the medial prefrontal cortex was more active when both the participant and another agent believed an object to be at a specific location, the right temporo-parietal junction was selectively activated during tracking the false beliefs of another agent about the presence, but not the absence of objects. While humans can explicitly attribute to a conspecific any possible belief they themselves can entertain, implicit belief tracking seems to be restricted to beliefs with specific contents, a content selectivity that may reflect a crucial functional characteristic and signature property of implicit belief attribution.

## Introduction

To successfully participate in social interactions, one must take into account that people are guided by mental states, such as desires and beliefs. Such “theory of mind” (ToM) abilities allow us to predict and interpret others’ behavior based on attributed mental states. Remarkably, human adults can attribute to another person any possible mental state that they themselves can hold, ranging from a belief about the location of an object, to more complex ones that, for instance, a juror may have when inferring criminal intent. ToM sometimes involves explicit and verbally expressed reasoning about mental states, but it could also operate implicitly and automatically without much deliberation.

According to a recent proposal the implicit ToM system employs different representations than does the explicit system [1]. Such ‘two-system’ approaches assume that automatic ToM relies on cognitive processes that are distinct from those employed by explicit mechanisms that are manifested in judgments of veridicality of others’ beliefs. In this view, only the latter can be considered proper ToM, while the implicit system is considered as a precursor. Alternatively, it was argued that implicit mental attributions reflect proper ToM, and their fast and efficient mechanisms may be crucial for real-life interactions from early on [2]–[4]. However, while there is extensive behavioral and neuroimaging research on explicit ToM, the functional properties and the underlying neural mechanisms of implicit ToM are less clear.

Neuroimaging research targeting explicit ToM reasoning has provided extensive evidence suggesting that a consistent set of brain regions is recruited when participants are required to reason about other people. This brain network (also termed social brain network or mentalizing network) includes the medial prefrontal cortex (MPFC), the bilateral temporo-parietal junction (TPJ), the superior temporal sulcus (STS), precuneus (PC) and the temporal poles [5]–[11]. In particular, two brain areas within the social brain network have been claimed to be crucial for ToM, namely the TPJ and MPFC. These brain areas are assumed to have well defined roles in reasoning about other people’s mental states. Specifically, Frith & Frith [7] have argued that the MPFC is involved in decoupling mental states from physical state representations and according to Saxe [12] the right TPJ is selectively involved in reasoning about other’s representational mental states.

However, most neuroimaging studies investigating ToM have employed paradigms following the standard false belief tasks, which require off-line deliberate reasoning and explicit and often verbal predictions based on mental states. A few investigations have used online or implicit tasks that elicited attributing goals to other agents, perspective taking or involved moral judgments, and reported the involvement of the social brain network in these tasks, such as the MPFC, or the TPJ, PC and STS regions [13]–[16]. Studies have also implemented methods where participants had an apparently unrelated task, but the situation could implicitly elicit thinking about other people. For instance, presentations of static natural scenes containing people while making simple category judgments (e.g., animal/vegetable) activated parts of the mentalizing network, specifically, the dorsomedial PFC and temporal poles [17]. Other studies, using online virtual reality tasks such as driving a taxi, found an increased activity in the right posterior STS, MPFC and right temporal pole for the events participants reported offline that they were engaged in thinking about other people [18]. Further investigations have addressed the question whether spontaneous trait inferences recruit the same brain networks as intentional inferences, revealing the involvement of the MPFC for both [19]. While most of these paradigms targeting implicit social cognition required attributing goals, traits or intentionality, rather than attributing representational mental states (i.e., beliefs) to other agents, in the present study we use a paradigm that directly taps on spontaneous computations concerning an agent’s false beliefs.

In a recent behavioral study investigating automatic ToM mechanisms, Kovács et al. [4] found that adults spontaneously tracked an agent’s belief about a location of an object, even when the agent and his beliefs were completely irrelevant for their task. The participants’ task was to detect the presence of an object, and their own belief that the object was present at a target location facilitated their performance. Importantly, object detection was also speeded when an additional observer, based on the perceptual input that was accessible for him, could have entertained the belief that the object was present, even though participants later observed the object having left the scene.

Two aspects of these results deserve closer attention. First, facilitation occurred without the instruction to encode the observer’s belief. This suggests that tracking the epistemic states of others, just like tracking others’ behavior in joint action [20] may be automatic [21]. Second, the above study of Kovács et al. [4] found asymmetric effects: While the detection of the object was facilitated by the false belief of the other observer that the object was present, the observer’s belief about the opposite state of affairs (i.e., that the ball was absent) did not interfere with object detection. Such an asymmetry might have been due to task demands, which required participants to respond only to the presence of the target, but not to its absence. However, it is also possible that this asymmetry is a functional characteristic of the implicit belief tracking system, which leads to preferential encoding of certain types of belief contents, while ignoring others in specific situations. The implicit ToM system may be specialized to track false beliefs about the presence, but not about the absence of objects.

In the current study we investigate implicit ToM by using functional MRI and a literature-based region of interest (ROI) approach. If automatic belief tracking recruits the same representational systems as explicit judgments, we expect that core brain regions previously reported to be active for explicit ToM tasks (i.e. MPFC and TPJ) to be also active in an implicit ToM task. Furthermore, by measuring brain activation during implicit belief tracking we can investigate whether the asymmetric sensitivity to false beliefs about the presence, but not the absence of objects reflects a genuine content-selectivity of the implicit system. We reasoned that if brain regions that are known to reflect belief attribution are active when an observer should think that an object is at a location (though it is not), and are not active when the observer should think that the object is not at a location (though it is), it would be evidence for the claim that automatic ToM tracks only specific kinds of beliefs (that is, beliefs with positive content, e.g., object at location, but not with negative content, e.g., object not at location).

## Materials and Methods

We recorded BOLD signal while participants were lying in the MRI scanner watching short movies, in which the movements of an agent, an occluder, and a ball were arranged to give rise to various potential belief contents to the agent. Like in the original study by Kovács et al. [4], participants were not required to monitor the agent’s beliefs. However, unlike in the original study, they had to respond to both the presence and the absence of the ball (eliminating the asymmetry of task demands).

### Participants

Fifteen healthy students (6 male; age: mean = 21.6, ranging from 18 to 27) participated on the basis of written informed consent. The study was conducted according to the Declaration of Helsinki, with approval of the local ethics committee of the University Hospital Gent. All subjects had normal or corrected-to-normal vision. No subject had a history of neurological, major medical, or psychiatric disorder. All participants were right-handed as assessed by the Edinburgh handedness questionnaire (mean score = 92).

### Design and stimuli

Participants were lying in the MRI scanner while watching short videos via a mirror. We have adapted the design and stimuli from Experiment 1 of Kovács et al. [4] with the modifications described below. We have used the same movies, except that they were 25% faster in this study, and we introduced a variable jitter between the two phases. All movies consisted of two phases: the belief formation phase and the outcome phase. Since we were specifically interested in the neural correlates of implicit belief formation, we introduced a variable jitter interval of 2, 3.5, 5, 6.5, 8, or 9.5 seconds between the two phases.

The movies in the belief formation phase differed along two aspects of the belief attributable to the agent: Content (Positive: ball present vs. Negative: ball absent) and Veridicality (True: matching reality vs. False: mismatching reality). Combined with the two versions of the outcome phase (ball does or does not appear from behind the occluder), there were 8 different trials, 6 jitter intervals, and movies were repeated twice during the study in a random order resulting in a total of 96 experimental trials. In addition, we inserted 12 null events consisting of a blank screen presented for the entire trial length.

#### Belief formation phase

**Figure 1:**
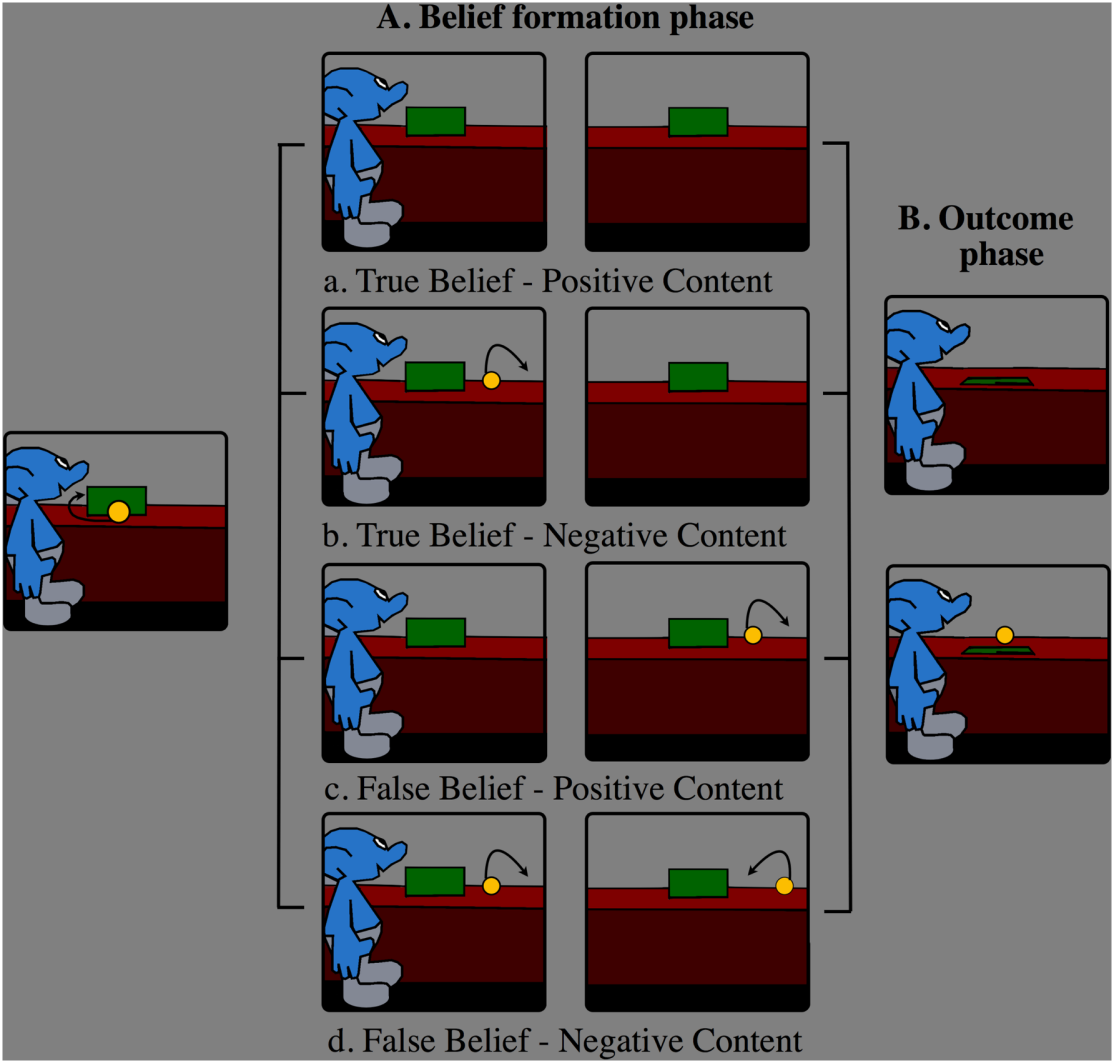
The logical structure of events in the experimental conditions. In the figure only the critical events are depicted, specifically, the final location of the ball and whether the agent was present or not when the event leading the outcome occurred (for the exact events and the timing see Methods).

As shown in Figure 1, all movies started with an agent placing a ball on a table in front of an occluder. Then the ball rolled behind the occluder. Following this, the movies could continue in four ways depending on the experimental conditions:

1. In the True Belief-Positive Content condition, the ball rolled out of the scene from behind the occluder, and then rolled back behind the occluder (ball last seen by the participant at 10 s; time information is given relative to the beginning of the movie) in the agent’s presence. The agent left the scene at 11 s. Thus, the agent could rightly believe the ball to be behind the occluder.
2. In the True Belief-Negative Content condition, the ball emerged from behind the occluder without leaving the scene, then rolled back behind the occluder, and finally left the scene (ball last seen at 10 s), all in the agent’s presence. The agent left the scene at 11 s. Thus, the agent could rightly believe the ball not to be behind the occluder.
3. In False Belief -Positive Content condition, we reversed the order of when the ball and the agent left the scene, respectively, relative to the True Belief –Negative Content condition. Thus, the agent left the scene at 6 s. Then, the ball emerged from behind the occluder without leaving the scene, rolled back behind the occluder, and finally left the scene (ball last seen at 11 s), all in the agent’s absence. Thus, the agent could wrongly believe the ball to be behind the occluder.
4. In the False Belief-Negative Content condition, the ball rolled out of the scene from behind the occluder in the agent’s presence. Then, the agent left the scene at 9 s. In his absence, the ball rolled back behind the occluder at 11 s. Thus, the agent could wrongly believe the ball not to be behind the occluder.

#### Outcome phase

At the end of each movie, the agent re-entered the scene and the occluder was lowered. The four conditions were paired with two outcomes, in which the ball was either present or absent behind the occluder. Participants were instructed to press one key when they detected the ball, and another key when they detected that the ball was not there (see Supporting Information, Additional analysis S1). Unlike in the Kovács et al. study [4], participants did not press a button when the agent left the scene, as we aimed to measure BOLD signal in the belief formation phase without possible movement artifacts. It is important to note that the required two alternative choice response (ball present/ball absent) in the outcome phase differed from that of Kovács et al. [4], where a detection (go-nogo) task was used rather than a choice response task. We changed the response in order to equate manual responses for ball presence and absence, and to make each outcome equally relevant. The ball was present in 50% of the trials in all conditions. Importantly, the agent’s beliefs were never mentioned and were irrelevant to the task. As we were interested in belief attribution processes, we restricted our analyses to the four conditions defined by the belief formation phase, independently of the outcome.

### MRI-Scanning Procedure

Images were collected with a 3T Magnetom Trio MRI scanner system (Siemens Medical Systems, Erlangen, Germany) using an 8-channel radiofrequency head coil. First, high-resolution anatomical images were acquired using a T1-weighted 3D MPRAGE sequence (TR = 2530 ms, TE = 2.58 ms, TI = 1100 ms, acquisition matrix = 256×256×176, sagittal FOV = 220 mm, flip angle = 7°, voxel size = 0.86×0.86×0.9 mm^3^). Whole brain functional images were collected using a T2*-weighted EPI sequence sensitive to BOLD contrast (TR = 2000 ms, TE = 35 ms, image matrix = 64×64, FOV = 224 mm, flip angle = 80°, slice thickness = 3.0 mm, distance factor = 17%, voxel size 3.5×3.5×3 mm^3^, 30 axial slices). Volumes aligned to AC-PC.

### FMRI analysis

The fMRI data were analysed with statistical parametric mapping using SPM5 software (Wellcome Department of Cognitive Neurology, London, UK). The first 4 volumes of all EPI series were excluded from the analysis to allow the magnetisation to approach a dynamic equilibrium. Data processing started with slice time correction and realignment of the EPI datasets. A mean image for all EPI volumes was created, to which individual volumes were spatially realigned by rigid body transformations. The high-resolution structural image was co-registered with the mean image of the EPI series. Then the structural image was normalised to the Montreal Neurological Institute (MNI) template, and the normalisation parameters were applied to the EPI images to ensure an anatomically informed normalisation. During normalisation the anatomy image volumes were resampled to 1×1×1 mm^3^. A filter of 8 mm FWHM (full-width at half maximum) was used. Low-frequency drifts in the time domain were removed by modelling the time series for each voxel by a set of discrete cosine functions to which a cut-off of 128 s was applied.

The subject-level statistical analyses were performed using the general linear model (GLM). The model contained separate regressors for all possible combinations of Veridicality (True vs. False), Content (Positive vs. Negative), phase (belief vs. outcome) and actual presence of the ball (present/absent) (duration of 0 seconds) resulting in 16 regressors in total. The percent signal change was extracted for the whole duration of the events of interest. Movement parameters were included to account for variance associated with head motion. All resulting vectors were convolved with the canonical haemodynamic response function (HRF) and its temporal derivative to form the main regressors in the design matrix (the regression model). The statistical parameter estimates were computed separately for each voxel for all columns in the design matrix.

The coordinates reported correspond to the MNI coordinate system.

#### Literature based ROIs in TPJ and MPFC

In order to obtain a ROI of TPJ and MPFC we conducted an activation-likelihood estimation (ALE) [22] meta-analysis on 26 studies on mentalizing that reported 31 peaks of activation in the proximity of TPJ and 31 in MPFC [23]. We used a threshold of FDR *p*<0.01 and a cluster size above 200 mm^3^. The cluster identified in TPJ was centred around the coordinate 56–47 33 (cluster size: 4448 mm^3^) and we used the mirrored ROI for the localization of left TPJ whereas the literature-based MPFC ROI was located at 2 53 13 (cluster size: 3368 mm^3^). Separately for each subject, each literature-based ROI, and each condition, the mean percent signal change over a time window of 4–13 s after stimulus onset was extracted (http://marsbar.sourceforge.net/) [24] and used for further analysis.

## Results

**Figure 2:**
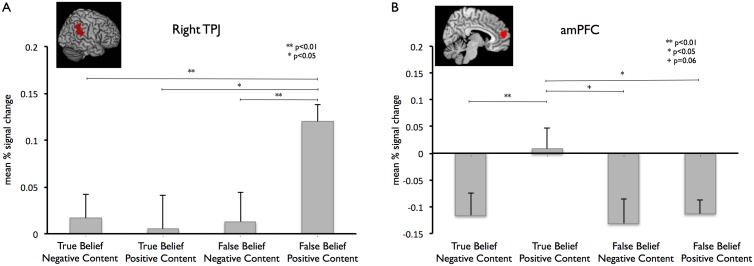
ROI mean percent signal change analysis for the right TPJ (A) and the amPFC (B).

We carried out signal-change analyses in the a-priori defined ROIs based on a meta-analysis of peaks reported in 26 studies on mentalizing. In right TPJ we found a main effect of belief (F(1,14) = 6.34, p = .025). Participants showed higher activation values for false than for true beliefs. Furthermore, there was a statistical trend for a main effect of content (F(1,14) = 4.37, p = .055). Importantly, a significant interaction effect of belief and content was found (F(1,14) = 5.35, p = .036) (Fig. 2A). Post-hoc t-tests revealed significant differences between the False Belief, Positive Content condition and all other conditions (True Belief, Negative Content: *t*(14) = −3.65, p<0.01; True Belief, Positive Content: *t*(14) = −2.64, p<0.05; False Belief, Negative Content: *t*(14) = −3.0, p<0.01).

In the left TPJ we did not observe any significant activation differences (Belief: F(1,14) = 0.22, p = .645, Content: F(1,14) = .784, p = .391, Belief*Content: F(1,14) = 1.09, p = .313). Furthermore, the signal-change analyses in the literature based MPFC ROI did not reveal any significant main effects (Belief: F(1,14) = 2.20, p = .160, Content: F(1,14) = 3.30, p = .091). Interestingly, however, it also showed a significant interaction of belief and content (Belief*Content: F(1,14) = 4.79, p = .046) (Fig. 2B). Post-hoc t-tests revealed significant differences between True Belief, Positive Content and True Belief, Negative Content (*t*(14) = −2.0, p<0.01) as well as False Belief, Positive Content (*t*(14) = −2.58, p<0.05), the difference to False Belief, Negative Content only revealed a tendency (*t*(14) = −1.98, p = 0.068).

## Discussion

The aim of the current study was to investigate two questions regarding the mechanisms and the underlying neural substrates of implicit belief tracking. One question was related to the neural mechanism of implicit ToM, and the other concerned the potential content selectivity of the implicit system. Regarding the first question, we found that implicit belief tracking, similarly to what is repeatedly found in studies targeting explicit ToM reasoning, recruits right TPJ and MPFC regions.

The finding that the right TPJ is only active when a false belief attributed to another person has a positive content reveals a crucial functional characteristic of the automatic belief tracking system, more specifically, a genuine content selectivity. This implies that spontaneous belief tracking may be initiated only for certain types of belief contents. When this content is about the occurrence of an object at a certain location, a positive content is attributed, while potential beliefs with negative content are ignored. One possible explanation for such pattern could be that this system may represent only false beliefs about the presence of an object (e.g., ‘he believes the ball is there’) because only these yield definite transitive action predictions related to the represented object, while false beliefs about the absence of an object (e.g., ‘he believes the ball is not there’) do not allow such predictions. Although we are certainly able to explicitly attribute to others any possible belief that we ourselves can entertain, including beliefs about the absence of objects (negative content), we conjecture that these might pose representational demands that the implicit system is not prepared to tackle.

Alternatively, such a limitation of the spontaneous belief tracking system may stem from the conflicting relation between the content of one’s own reality representation and that of an attributed belief. According to this possibility, one would spontaneously track someone else’s belief only when one does not have a strong competing own belief. Thus, in our case one would compute the belief of the agent in the condition where one believes nothing to be present behind the occluder, but not when one believes the ball to be behind the occluder. While it is difficult to separate the two alternatives with the current design, data from other studies with infants and adults seem to support the interpretation that the limitation may be related to the negative belief content. Indeed, both infants and adults seem to spontaneously track others’ beliefs and perspective even if these are strongly competing with their own representations [25], [10].

On the other hand, the MPFC was more active in the condition where both the participant and the agent believed that the ball was behind the occluder (true belief with a positive content). Recent studies have found that the MPFC is recruited in situations where an actor searches in a location where an object is present compared to an empty location [26]. While there was no explicit object search induced in the present task, we have also found a higher activation pattern in the condition where both the participant and the agent believed that the object was behind the occluder, thus allowing for a possible search. Furthermore, this higher MPFC activation pattern in the true belief- object present condition is also in line with proposals suggesting that the MPFC is involved in reasoning about triadic relations between Me, You and an object [12], but might not be selectively recruited for attributing representational mental states [27], [28]. Indeed, in the present study, using an implicit belief attribution task that is analogous to earlier used explicit ToM task, we found an activation of the MPFC in the true belief - object present condition, but not in the false belief conditions. Earlier studies have found that the MPFC is involved in representing various characteristics of other agents besides their beliefs, such as their appearance and emotions [27], [28] or a viewpoint-independent perspective selection [15].

While in the last years there seems to be more consensus on the selective role of the right TPJ in processing mental states with representational content [12], [27], [29], researchers have also proposed that right TPJ activity may not be selective for social cognition, as both ToM and attentional reorienting tasks were found recruit this area [30]. Additionally, research has shown that the TPJ and the MPFC are also associated with self-other distinction [23]. Our finding that implicit ToM seems to recruit the right TPJ is consistent with what is usually found using explicit ToM tasks, although the left TPJ might also play a role in ToM reasoning, as lesion studies have reported that damage to left TPJ is associated to deficits on explicit ToM tasks [31]–[33].

One might wonder whether the implicit vs. explicit distinction is warranted in ToM research, as it is unclear whether it refers to the nature of the task or to the underlying cognitive processes, and we concur with such worries. After all, one could argue that even in our study participants could have spontaneously engaged in explicit, besides implicit, mentalizing, even if they were not instructed to do so. However, if our participants recruited similar computations as the participants in the Kovács et al. [4] study, where equivalent belief tracking effects were found in adults and infants, than given that young infants are thought to lack an explicit belief tracking system, one could argue that our participants most likely have relied on their implicit ToM system as well. Additionally, according to standard views, explicit ToM, in contrast to implicit ToM, should be effortful, highly dependent on cognitive resources and occur offline [1]. However, since we measured the BOLD signal online as the belief scenario unfolded, we find it unlikely that participants could have engaged in explicit and effortful ToM processes. While we did not systematically debrief the participants in the present study, in the earlier Kovács et al. study participants reported that they had believed the agent to be irrelevant or that it was a mere distractor [4] (Supplementary Material, Additional analysis S1, p. 5).

Furthermore, regarding the issue of automaticity in mental state reasoning, earlier studies have found a modulation of the dorsomedial prefrontal cortex by cognitive load when participants were instructed to think of the reasons why a character might perform specific actions [34]. In a framework where automaticity is not seen a unitary construct but instead as comprising a set of relatively independent dimensions, such as efficiency, awareness, intention, and control [35], our study seems to speak mostly to the intention and awareness dimensions, as participants were not instructed to intentionally track the agent’s beliefs (and were likely not aware of doing so).

In summary, our findings suggest that the mechanisms underlying the automatic tracking of others’ beliefs exploit partly similar representational systems as explicit ToM judgments do. Furthermore, we have found evidence for a content-dependent representational constraint on implicit ToM, which restricts the system to tracking false beliefs that may allow fast and efficient predictions about others’ actions. Such a content-selectivity favoring potential behaviorally relevant beliefs may represent the signature limit of the implicit ToM system and may signal a functional difference between implicit and explicit ToM attributions.

## Supporting Information

Additional analysis S1 (PDF)
