# Supplementary material for: Are All Beliefs Equal? Implicit Belief Attributions Recruiting Core Brain Regions of Theory of Mind
Source: PLoS One. 2014 Sep 26;9(9):e106558. doi: 10.1371/journal.pone.0106558 (PMC4178016; doi:10.1371/journal.pone.0106558)
Supplement: Additional analysis S1 — (PDF) [file pone.0106558.s001.pdf]

Á. M. Kovács, S. Kühn, G. Gergely, G. Csibra, M. Brass

Supporting Information

Additional analysis S1

Behavioral task

The primary focus of the present study was investigating the belief formation phase (online belief tracking) via analyzing the BOLD signal (independently of the outcome phase). However, in order to keep participants attention in the scanner and to give them a task that was unrelated to the belief of the agent, in the outcome phase participants had to press one key if the ball was present, and another key when it was absent. Thus, unlike in Kovács et al. [4], here participants pressed a key at the end of each trial, to equate for manual choice responses between the conditions. For the reasons described above we decided to use a choice response task rather than a detection (go-nogo) task, as in Kovács et al. [4].

Furthermore, the procedure we used in the present study was also different in another important aspect from the procedure used by Kovács et al. [4]. In order to be able to measure the BOLD signal in the belief formation phase, we introduced a variable jitter period between the belief formation and the outcome phase varying between 2 to 9.5 seconds.

With these differences in mind, we performed an ANOVA on the reaction time responses with the factors Agent's belief (Ball present vs. Ball absent), Participant's belief (Ball present vs. Ball absent), and Outcome (Ball present vs. Ball absent). We have found a main effect of the Outcome $F(1,13) = 28.32$, $p = .0001$, and no other

effects or interactions. Participants were overall faster when they had to respond that the ball was there (mean RT = 301 ms), compared to the conditions when they had to respond that the ball was not there (mean RT = 347 ms; Bonferroni post hoc comparison p = .0001). The mean RTs were the following in the conditions where the ball was present in the outcome, reported separately for the 4 experimental conditions (in ms): True Belief-Positive Content =297 (SEM=29), True Belief-Negative Content =308 (SEM=31), False Belief-Positive Content =302 (SEM=31); False Belief - Negative Content =296 (SEM=29). In the conditions where the ball was absent in the outcome, the mean RTs (ms) were: True Belief-Positive Content =311 (SEM=31), True Belief-Negative Content =348 (SEM=28), False Belief-Positive Content =370 (SEM=37), False Belief -Negative Content =344 (SEM=32).

This response pattern is not surprising, as detecting the presence of an object is likely easier than detecting its absence. However, given the different response type used and the variable delay between the belief formation phase and the outcome phase these RTs are not comparable to the reaction time pattern found in Kovács et al. [4].
